# Character strengths as predictors of general and academic self-efficacy in university students

**DOI:** 10.3389/fpsyg.2024.1490095

**Published:** 2024-12-04

**Authors:** Diego García-Álvarez, Rubia Cobo-Rendón, Karla Lobos

**Affiliations:** ^1^Departamento de Ciencias del Comportamiento, Universidad Metropolitana, Caracas, Venezuela; ^2^Centro de Estudios de Psicología, Universidad de Montevideo, Montevideo, Uruguay; ^3^Instituto de Bienestar Socioemocional (IBEM), Facultad de Psicología, Universidad del Desarrollo, Concepción, Chile; ^4^Escuela de Psicología, Facultad de Educación y Ciencias Sociales, Universidad Andrés Bello, Concepción, Chile

**Keywords:** general self-efficacy, academic self-efficacy, character strengths, university students, positive psychology

## Abstract

Positive psychology has introduced the concept of character strengths, which are positive traits fundamental to well-being and mental health. Research on university students has shown that these strengths impact psychoeducational variables and personal functioning, acting as a protective factor in the general and student populations. This study aims to analyze the predictive relationships between character strengths and general self-efficacy and determine their joint contribution in predicting academic self-efficacy. The study was quantitative, correlational-causal, and cross-sectional, using a non-probabilistic sample of 668 Venezuelan university students (68.86% women, average age of 20.52 years). The scales used are Growing Strong to measure character strengths, the General Self-Efficacy Scale, and the Self-Efficacy Scale in Specific Academic Situations. All scales showed adequate psychometric properties. The mediation analysis revealed that leadership, hope, and persistence positively influence general and academic self-efficacy. Furthermore, general self-efficacy strongly impacts academic self-efficacy. In conclusion, character strengths are significant predictors of self-efficacy in university students. Promoting these strengths may be crucial to improving general and academic self-efficacy, suggesting the need to implement specific initiatives in higher education.

## 1 Introduction

Traditional psychology has devoted much of its efforts to understanding and treating human suffering. While this approach has been fundamental, it needs to examine the study of positive experiences and individual strengths. Positive psychology emerges as a response to this need, proposing a more comprehensive approach to healing wounds and cultivating people’s capacities and potential, emphasizing the construction of competencies and health promotion. Recently, there has been an emphasis on building well-being in cultural and linguistic contexts (Lomas et al., 2021).

Within the theoretical framework of positive psychology, character strengths emerge as fundamental elements for promoting well-being. Peterson and Seligman (2004) defined strengths as patterns of thought, affect, and behavior that contribute to a fulfilling life. These innate qualities, which develop through the pursuit of personal values (Park et al., 2004), enable us to face life’s challenges with resilience and significantly contribute to our overall well-being (De la Fuente et al., 2022). Indeed, these strengths can be considered the foundational pillars of our virtues (Park et al., 2006).

Studies conducted before and during the pandemic have identified character strengths as psychological resources related to life satisfaction, both subjective and physical well-being (Proyer et al., 2013). They also indicate that strengths facilitate better life transitions during adversity and are essential for coping, resilience, adaptive behaviors, personal growth, and mental health (Waters et al., 2022). Specific research with university students has found that character strengths are associated with life satisfaction (Allan et al., 2021), as well as flourishing (Chan et al., 2022), subjective well-being, and psychological well-being (Azañedo et al., 2020), along with coping flexibility (Ferradás-Canedo et al., 2021). Conversely, research shows that strengths are associated with lower levels of stress, (Kaya, 2023; Uliaszek et al., 2022), emotional distress (García-Álvarez et al., 2023), negative affectivity, and neuroticism (Anjum and Amjad, 2021), as well as depressive symptoms (Yu et al., 2023). Thus, research has shown relationships between character strengths and mental health variables in university students.

Exploring students’ university experience reveals interesting associations between character strengths and various psychoeducational variables, suggesting positive relationships with successful educational trajectories. These include student persistence (Browning et al., 2018), academic performance (Cosentino and Solano, 2012), academic achievement, and college success (Golding et al., 2018), GPA scores (Bachik et al., 2021; Lounsbury et al., 2009), time spent studying, and satisfaction with learning (Littman-Ovadia and Freidlin, 2022), as well as career adaptability (Magnano et al., 2021). In contrast, character strengths have negative relationships between academic procrastination (García-Álvarez et al., 2023) and professional doubt (Villacís-Nieto and Naval, 2021).

Research on character strengths in the university context has also addressed interventions to enhance positive outcomes. In this regard, Dolev-Amit et al. (2021) reported positive results regarding the effectiveness of a strength-focused intervention in improving optimism and reducing levels of negative affect and psychological distress. Research by Yu et al. (2023) demonstrated that an intervention focused on awareness, exploration, and application of character strengths could be an effective tool for improving psychological well-being, especially during crises such as the COVID-19 pandemic, suggesting that this intervention could be a viable alternative to traditional counseling.

Additionally, Green (2024) reported positive results from a character strengths-based intervention that increased personal growth initiative among university students. Similarly, Zammitti et al. (2023) reported the effectiveness of a multi-component intervention based on Positive Psychology and Life Design to support the transition of university students into the workforce, explicitly indicating significant improvements in psychological resources such as resilience, career adaptability, hope, and self-efficacy after training. This evidence suggests that such programs can be highly beneficial in helping young people adapt to current challenges and build a satisfying professional future.

Character strengths also relate to cognitive constructs associated with self-perception in university students. Researchers have linked these strengths to personal value judgments, including self-esteem (Dolev-Amit et al., 2021; Douglass and Duffy, 2015; García-Álvarez et al., 2020; Huber et al., 2017; Macaskill and Denovan, 2014). Additionally, evidence with adolescent students has indicated more significant relationships between the following strengths with general self-efficacy (showing coefficients ≥0.50): hope, perspective, creativity, vitality, teamwork, social intelligence, and gratitude (Ruch et al., 2014). Studies on university students show that strengths and strengths use (Proctor et al., 2011) positively correlated with self-efficacy (García-Álvarez et al., 2020). Social Cognitive Theory of Bandura’s (2012) defines self-efficacy as students’ beliefs in their abilities to perform tasks at specific levels of competence (Bandura, 2012). These beliefs significantly influence the events that shape their lives, and some scholars argue that general self-efficacy represents a broad competence for coping with life’s challenges (Baessler and Schwarzer, 1996). Research indicates that general self-efficacy is crucial for educational success, mental health, and overall adjustment among university students (Lin et al., 2023; Manzano-Sanchez et al., 2018).

Despite extensive research on self-efficacy, Díaz-Mujica et al. (2022) have pointed out various methodological issues in their systematic review of this construct in university students. They highlight conceptual confusion and measurement inconsistencies. For instance, researchers note that they often evaluate specific self-efficacy measures in general terms and vice versa. They also identify inconsistencies in using other scales related to self-regulated learning and attitudes toward learning, which assess academic self-efficacy. These findings emphasize the need to differentiate and clarify the construct of self-efficacy in research. Bandura (2012) argues that researchers should analyze this construct based on activity domains and situational specifics as it manifests at different levels. In the academic context, beliefs about one’s abilities, competencies, and resources are considered academic self-efficacy (Dominguez-Lara, 2016).

Only some studies have addressed this differentiation. Blanco (2010) also highlighted this methodological difficulty in the Spanish university context, confirming that self-efficacy beliefs specific to the academic domain are distinguished from other self-referential constructs, such as general self-efficacy. Montes De Oca and Moreta-Herrera (2019) reported a strong, positive, and significant relationship between both constructs; as one variable increases, so does the other. Furthermore, regression analysis found that general and academic self-efficacy constructs did not present multicollinearity issues, including both as predictors in models of academic motivation increased explanatory capacity. This finding is consistent with what García-Álvarez (2024) reported, highlighting the predictive nature of general self-efficacy over specific self-efficacy in academic situations for university students.

Focusing on the relationships between character strengths and academic self-efficacy in German secondary students, researchers found high correlations with 21 out of 24 strengths, particularly perspective, hope, love of learning, perseverance, vitality, bravery, and social intelligence (Weber and Harzer, 2022). In contrast, all character strengths strongly correlated with academic self-efficacy in Filipino students, with the strength of hope predicting higher levels through regression analysis (Datu and Mateo, 2020). The evidence presented regarding the relevance of character strengths and self-efficacy, such as general and academic constructs associated with educational performance, well-being, and mental health variables in university students, invites a deeper exploration of the relationship between character strengths and general and academic self-efficacy. The evidence is crucial, as they are distinct psychological constructs that refer to beliefs in one’s abilities to cope with various situations in everyday life and the academic realm. From a practical perspective, this paper can guide professionals on which character strengths to prioritize in interventions within educational settings. Identifying and promoting these strengths can serve as an effective strategy for enhancing students’ general and academic self-efficacy. The three constructs—character strengths, general self-efficacy, and academic self-efficacy—demonstrate significant benefits in university students’ mental health and psychoeducational functioning. This research not only enhances the theoretical understanding of their interrelationship but also provides a practical framework for developing educational programs that promote students’ overall well-being. In this regard, strengthening these qualities can enhance personal and professional growth as students face challenges throughout their educational journey.

The evidence presented in the introduction suggests that specific character strengths, such as perseverance, hope and social intelligence, relate significantly to general and academic self-efficacy. Previous research has highlighted that these specific strengths notably impact students’ self-efficacy. Additionally, studies show that general self-efficacy directly influences academic self-efficacy; students who trust their ability to handle stressful situations tend to demonstrate greater confidence in their capacity to tackle specific academic tasks, this increases motivation and persistence in their studies (Covarrubias-Apablaza et al., 2019). However, it is essential to identify which specific character strengths influence both dimensions of self-efficacy. Given the broad spectrum of 24-character strengths, it is reasonable to expect that not all will have the same impact in specific academic contexts. Therefore, this study will focus on strengths that significantly correlate with general and academic self-efficacy. This choice will not only enable the development of more effective and targeted educational interventions but also help validate and enrich the study’s conceptual framework. By demonstrating how specific strengths directly impact self-efficacy, this research will contribute to the existing body of knowledge on positive psychology. This research aims to analyze the predictive relationships between character strengths and general self-efficacy and, in turn, to determine the joint contribution of these two variables in predicting academic self-efficacy in university students. Based on the evidence presented, we propose the following hypothesis: Character strengths positively influence academic self-efficacy in university students, and general self-efficacy mediates this relationship.

## 2 Method

### 2.1 Participants

The sample comprised 668 students aged 18 to 36 (M = 20.52, SD = 2.27), all of them enrolled in the Psychology degree at several private universities in Venezuela. The sample included participants from four academic years, with one course per academic year to approximate representation from each level of the program. Four hundred sixty female students (68.9%) and 208 male students (31.1%) were surveyed. The distribution of the 668 students by academic year and gender reveals a trend of greater female participation compared to male participation in each academic year. In the first year, of the 235 students, 155 are women and 80 men, representing 65.96 and 34.04% of the total for that year, respectively. In the second year of the 238 students, 172 are women and 66 men, which constitutes 72.27 and 27.73% of the annual total. The third year has 123 students, where 91 are women and 32 men, representing 73.98 and 26.02% of the total for the year, respectively. Finally, in the fourth year of the 72 students, 42 are women and 30 men, with percentages of 58.33 and 41.67%, respectively.

An intentional non-probabilistic sampling method was used based on student availability. The selection criteria were as follows: (1) psychology students enrolled on a regular basis during the measurement period, (2) from any academic level, (3) of both sexes, (4) present on campus during the measurement period, and (5) willing to participate in the study.

### 2.2 Instruments

#### 2.2.1 Character strengths

The Growing Strong scale is a scale to assess character strengths. It is a measure of South American origin designed in Spanish to evaluate the construct locally. It is a Likert scale made up of 48 items with five response options, this is an example item “there are other ways to see things or understand things that are different from my own”; it evaluates the 24-character strengths, it has presented adequate psychometric properties in both reliability and validity in the Venezuelan population, it adjusts to the factorial structure of six virtues proposed by Peterson and Seligman (2004): χ^2^/gl = 2, CFI = 0.95, TLI = 0.95, SRMR = 0.057, RMSEA = 0.032, with reliability measured by Cronbach’s Alpha = 0.920, (García-Álvarez et al., 2020). In this study, we investigated the factorial structure of the instrument using this sample. We confirmed the grouping of six virtues, with adequate indicators including χ^2^/gl = 3.7, CFI = 0.96, TLI = 0.95, SRMR = 0.07, and RMSEA = 0.06. Additionally, we measured the reliability of the factors using Cronbach’s Alpha, obtaining values that ranged from 0.69 to 0.84.

#### 2.2.2 General self-efficacy

The Spanish version of the eight-item General Self-Efficacy Scale by Baessler and Schwarzer (1996) is a Likert scale with five response options, this is an example item “I am confident that I could deal efficiently with unexpected events.” In Venezuela, it has shown adequate psychometric properties at the level of validity and reliability, evaluating general self-efficacy in a unifactorial way: χ^2^/gl = 5, CFI = 0.99, TLI = 0.99, SRMR = 0.03, RMSEA = 0.10, with reliability measured by Cronbach’s Alpha = 0.92, (García-Álvarez et al., 2022). In this study, we confirmed the unifactorial structure using the current sample, obtaining adequate indicators: χ^2^/gl = 3.6, CFI = 0.99, TLI = 0.99, SRMR = 0.03, and RMSEA = 0.06. The reliability measured in this study was Cronbach’s Alpha = 0.90.

#### 2.2.3 Academic self-efficacy

The Perceived Self-Efficacy Scale Specific to Academic Situations was designed by Palenzuela (1983) in its adaptation for Latin America by Dominguez-Lara (2016) in its seven-item version. It is a Likert scale with five response options, this is an example item “I believe I have the ability to understand a subject well and quickly.” The scale assesses academic self-efficacy in a unifactorial way. In Venezuela, it has shown adequate psychometric properties at the level of validity and reliability: χ^2^/gl = 3.43, CFI = 0.99, TLI = 0.99, SRMR = 0.04, RMSEA = 0.07, with reliability measured by Cronbach’s Alpha = 0.88 (García-Álvarez et al., 2022). In this study, we confirmed the unifactorial structure using the current sample, obtaining adequate indicators: χ^2^/gl = 2.2, CFI = 0.99, TLI = 0.99, SRMR = 0.03, and RMSEA = 0.04. The reliability measured in this study was Cronbach’s Alpha = 0.83.

### 2.3 Procedure

This study is part of an extensive investigation titled Character Strengths, general and Academic Self-efficacy in university-level students: starting point for psychoeducational intervention, evaluated by the Research and Development Division of the Metropolitan University, Venezuela. The study was quantitative, correlational-causal, and cross-sectional. The study followed the ethical guidelines of the Declaration of Helsinki, the American Psychological Association, and the Venezuelan Federation of Psychologists. To collect data in an online form, the students had an informed consent explaining the study’s objective, referring to anonymity, confidentiality, and safeguarding of the data, and ensuring that they did not present risks to the participants’ mental health.

### 2.4 Data analysis plan

Before moving on to the descriptive results and correlations, we examined the psychometric properties of each instrument. We conducted confirmatory factor analyses (CFA). We assessed the validity of the scales using the Diagonally Weighted Least Squares (DWLS) estimator due to the specific characteristics of the data and scales employed. We used fit indices such as the Comparative Fit Index (CFI), Tucker-Lewis Index (TLI), Root Mean Square Error of Approximation (RMSEA), and Standardized Root Mean Square Residual (SRMR), interpreting these indices according to the criteria established by Hu and Bentler (1999). These criteria suggest that CFI and TLI values greater than 0.95 indicate optimal fit, while RMSEA and SRMR values below 0.08 indicate an acceptable model fit. Additionally, we considered Cronbach’s Alpha to evaluate the internal reliability of the scales, thereby ensuring the consistency of the instruments used in the study. Descriptive and correlational analyses were conducted to explore the relationship between character strengths and general and academic self-efficacy in college students, using all the responses obtained in the data collection process, with no missing data in the analysis. The analyses met the assumptions of normality, linearity, and homoscedasticity. Mediation analyses allowed us to determine the joint contribution of these variables in the prediction of academic self-efficacy, assuming independence and normality of the errors, a theoretical basis for the causal relationship and the absence of multicollinearity. This approach allowed decomposing the total effect into direct and indirect effects, providing a more precise view on the role of character strengths and self-efficacy in the academic context.

## 3 Results

This study aimed to analyze how character strengths and general self-efficacy predict academic self-efficacy in university students. The results showed that the mean scores for character strengths indicated moderately high levels among the students, with Gratitude being the most prominent strength. The distributions of the variables were skewed towards higher scores, with negative skewness and elevated kurtosis, particularly in strengths such as Appreciation of Beauty and Gratitude, indicating that many students scored near the high average value in these strengths (See Table 1).

**Table 1:** Descriptive statistics for character strengths, general self-efficacy, and academic self-efficacy.

Variable	Mean	Standard deviation	Skewness	Kurtosis
Creativity	8.356	1.434	−1.254	2.499
Curiosity	8.877	1.113	−1.459	4.667
Open-mindedness	8.769	1.222	−1.568	4.607
Love of learning	9.216	1.175	−2.549	9.874
Perspective	8.840	1.186	−1.462	4.249
Honesty	8.594	1.411	−1.056	1.180
Bravery	8.204	1.522	−0.951	1.308
Persistence	8.543	1.330	−1.254	2.313
Vitality	7.052	1.949	−0.385	−0.416
Kindness	8.726	1.240	−1.577	4.948
Love	8.985	1.363	−2.312	7.687
Social intelligence	7.975	1.612	−0.987	1.414
Fairness	9.135	1.122	−2.783	12.452
Leadership	7.049	1.994	−0.321	−0.498
Teamwork	8.147	1.466	−0.742	0.669
Forgiveness	8.105	1.559	−0.937	1.240
Modesty	8.499	1.327	−1.255	3.050
Prudence	7.934	1.479	−0.645	0.538
Self-regulation	7.728	1.588	−0.770	0.668
Appreciation of beauty and excellence	9.075	1.182	−2.160	7.335
Gratitude	9.238	1.118	−2.470	9.700
Hope	8.545	1.521	−1.417	2.771
Humor	8.582	1.352	−1.187	2.344
Spirituality	8.825	1.487	−1.633	3.170
Academic Self-Efficacy	25.470	5.057	−0.382	−0.615
General Self-Efficacy	29.696	6.349	−0.284	−0.731

Standard Error of Skewness = 0.095; Standard Error of Kurtosis = 0.189.

### 3.1 Correlations of character strengths with general and academic self-efficacy

To explore the relationships between character strengths and general and academic self-efficacy, a Pearson correlation analysis was conducted, finding that all correlations were statistically significant (*p* < 0.001). Among the strengths related to general self-efficacy were persistence, hope, and social intelligence (*r* = 0.41 to 0.31). In the academic context, the most prominent strengths were hope, leadership, and persistence (*r* = 0.45 to 0.24). The strengths with the highest correlations in both domains, such as hope, persistence, and leadership, stand out for their relations on the perception of self-efficacy. Additionally, social intelligence, humor, and creativity also show a strong relationship with both general and academic self-efficacy, highlighting their relevance in developing robust self-efficacy in various contexts (Table 2).

**Table 2:** Correlations of character strengths with general self-efficacy and academic self-efficacy.

Character strength	r with general self-efficacy	r with academic self-efficacy
Creativity	0.344***	0.244***
Curiosity	0.213***	0.240***
Open-mindedness	0.290***	0.244***
Love of learning	0.154***	0.173***
Perspective	0.243***	0.242***
Honesty	0.261***	0.210***
Bravery	0.333***	0.214***
Persistence	0.411***	0.367***
Vitality	0.309***	0.269***
Kindness	0.220***	0.168***
Love	0.153***	0.144***
Social intelligence	0.368***	0.309***
Fairness	0.201***	0.170***
Leadership	0.341***	0.376***
Teamwork	0.357***	0.290***
Forgiveness	0.240***	0.211***
Modesty	0.252***	0.201***
Prudence	0.323***	0.208***
Self-regulation	0.295***	0.203***
Appreciation of beauty and excellence	0.268***	0.251***
Gratitude	0.242***	0.236***
Hope	0.410***	0.446***
Humor	0.341***	0.324***
Spirituality	0.247***	0.310***

***p < 0.001.

### 3.2 Mediation of general self-efficacy in the relationship between character strengths and academic self-efficacy

Table 3 presents the direct, indirect and total effects of character strengths (hope, persistence and leadership) on academic self-efficacy, considering general self-efficacy as a mediator. The results indicate that hope has a significant direct effect on academic self-efficacy (β = 0.127, *p* < 0.001), as well as a significant indirect effect through general self-efficacy (β = 0.074, *p* < 0.001), resulting in a significant total effect (β = 0.200, *p* < 0.001). Similarly, persistence presents a non-significant direct effect on academic self-efficacy (β = 0.015, *p* = 0.562), but a significant indirect effect through general self-efficacy (β = 0.084, *p* < 0.001), resulting in a significant total effect (β = 0.099, *p* < 0.001). Finally, leadership shows both a significant direct effect (β = 0.075, *p* < 0.001) and a significant indirect effect (β = 0.052, *p* < 0.001), leading to a significant total effect (β = 0.127, *p* < 0.001).

**Table 3:** Direct, indirect, and total effects of hope, persistence, and leadership on academic self-efficacy.

Character strength	Type of effect	Estimate	Standard error	z-value	*p*-value	IC 95% lower–upper
Hope	Direct	0.127	0.023	5.518	<0.001	0.082–0.172
	Indirect	0.074	0.014	5.343	<0.001	0.047–0.100
	Total	0.200	0.026	7.751	<0.001	0.150–0.251
Persistence	Direct	0.015	0.026	0.580	0.562	−0.036–0.067
	Indirect	0.084	0.016	5.330	<0.001	0.053–0.115
	Total	0.099	0.030	3.350	<0.001	0.041–0.157
Leadership	Direct	0.075	0.016	4.860	<0.001	0.045–0.106
	Indirect	0.052	0.009	5.581	<0.001	0.034–0.070
	Total	0.127	0.017	7.323	<0.001	0.093–0.161

In terms of variance explained, the R^2^ for general self-efficacy was R^2^ = 0.260, indicating that the model explains 26% of the variance in general self-efficacy from character strengths. For academic self-efficacy, the R^2^ reached a value of R2 = 0.457 in the full model that includes both the direct effects of character strengths and the mediating effect of general self-efficacy. This indicates that the model manages to explain 45.7% of the variance in academic self-efficacy. The inclusion of general self-efficacy as a mediator contributed to an additional 19.7% increase in the explained variance in academic self-efficacy, highlighting its role as a key mediator.

**Figure 1:**
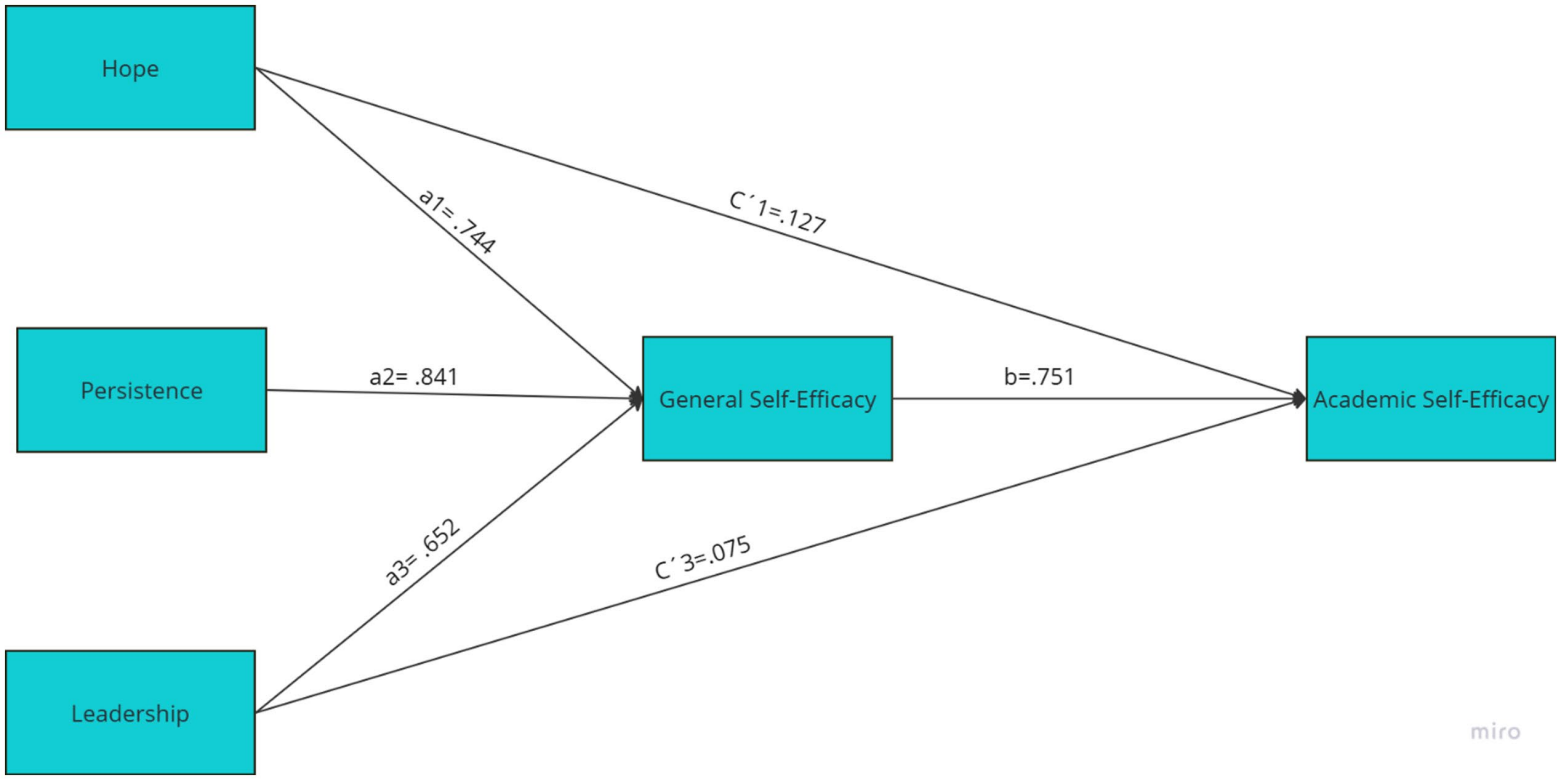
Mediation model of general self-efficacy in the relationship between the strengths of hope, persistence, and leadership with academic self-efficacy. a1, a2, and a3 = coefficients of the relationships of hope, persistence and leadership on General Self-efficacy respectively; b = effect of general self-efficacy on academic self-efficacy; C′1 and C′3 = coefficients of the direct effects of the Hope and Leadership variables (respectively) on Academic Self-Efficacy, adjusting for the effect of General Self-Efficacy.

In terms of total effects, hope (β = 0.200, *p* < 0.001), persistence (β = 0.099, *p* < 0.001), and leadership (β = 0.127, *p* < 0.001) were significant, indicating that these strengths positively impact academic self-efficacy, with partial mediation by general self-efficacy (see Figure 1). These findings suggest that general self-efficacy partially mediates the relationship between these character strengths and academic self-efficacy, highlighting the importance of these strengths in fostering self-efficacy in educational contexts.

## 4 Discussion

The results of this study offer a comprehensive insight into the predictive relationships between character strengths and self-efficacy, both general and academic, among university students. We found that character strengths such as hope, persistence, and leadership significantly predict general and academic self-efficacy in university students. Moreover, we discovered that general self-efficacy mediates the relationship between these character strengths and academic self-efficacy. These findings underscore the importance of understanding and promoting these strengths in the university context, as their influence on self-efficacy has significant implications for students’ academic performance and well-being.

### 4.1 Correlations of character strengths with general and academic self-efficacy

Regarding correlations, the results reveal significant relationships between various character strengths and both general and academic self-efficacy. These results are consistent with previous research reporting correlations between character strengths and general self-efficacy in adolescent students (Ruch et al., 2014) and university students (Proctor et al., 2011; García-Álvarez et al., 2020). Similarly, other studies have found positive and significant correlations between most character strengths and academic self-efficacy in cross-sectional (Weber and Harzer, 2022) and longitudinal research involving students (Datu and Mateo, 2020). Overall, these results support Peterson and Seligman (2004) claims about the link between good character, expressed through character strengths, and positive outcomes, particularly regarding self-efficacy. These strengths correlate with beliefs in one’s ability to organize and execute practical actions, significantly affecting thoughts, feelings, and behaviors and impacting performance in general life contexts and specific situations like academics (Bandura, 2012). The strengths that exhibited the highest correlations with general self-efficacy were persistence, hope, and social intelligence, consistent with the previously cited studies. Researchers interpret these results from the theoretical perspective that persistence drives individuals to maintain effort and interest in achieving long-term goals despite difficulties and obstacles. Persistent individuals demonstrate tenacity and continue striving until they reach their objectives. In contrast, hope involves believing in one’s ability to positively influence the future, which fosters a greater sense of self-efficacy. Finally, practical leadership —the ability to manage interpersonal relationships well—enhances self-efficacy by reinforcing confidence in one’s abilities through feelings of competence in social situations.

On the other hand, the strongest correlations with academic self-efficacy were found in strengths such as hope, leadership, and persistence. These results highlight these strengths’ role in the academic field, where confidence in achieving educational goals is vital for academic success. Hope has been identified in previous research as a critical predictor of resilience and perseverance in educational contexts, which is consistent with the findings of this study (Arias et al., 2020). Additionally, significant positive correlations between strengths such as creativity, curiosity, and open-mindedness with academic self-efficacy suggest that innovative, inquisitive students who are open to new ideas tend to feel more capable and confident when facing academic challenges. This confidence stems from their ability to approach problems from multiple angles, constant desire to learn, and willingness to consider diverse perspectives, which is consistent with various studies that report the importance of creativity-related skills in academic success (Abad-Segura and González-Zamar, 2019; De La Cruz Velazco et al., 2022).

### 4.2 Mediation of general self-efficacy in the relationship between character strengths and academic self-efficacy

These results are consistent with existing literature linking character strengths to well-being, self-cognitions, and variables related to academic performance (Bachik et al., 2021; Bandura, 2012; Browning et al., 2018; Chan et al., 2022; Cosentino and Solano, 2012; Dolev-Amit et al., 2021; Golding et al., 2018; Littman-Ovadia and Freidlin, 2022; Lounsbury et al., 2009; Park et al., 2004). The mediation observed by general self-efficacy indicates that while strengths such as hope, leadership, and persistence directly impact academic self-efficacy, their influence is amplified when students also possess high general self-efficacy (García-Álvarez et al., 2020; Proctor et al., 2011; Ruch et al., 2014; Weber and Harzer, 2022). In line with previous studies, general self-efficacy significantly influences academic self-efficacy in specific situations (Bandura, 2012; Blanco, 2010; Covarrubias-Apablaza et al., 2019; García-Álvarez, 2024; Montes De Oca and Moreta-Herrera, 2019). When individuals believe in their ability to overcome challenges in various areas, this confidence translates into a stronger sense of self-efficacy academically. This connection allows students to feel more prepared to tackle tasks, manage exam stress, and engage actively in their learning, enhancing their academic performance. Also, these findings suggest that developing strong general self-efficacy may be an essential strategy for enhancing perceptions of self-efficacy in educational contexts. Promoting strengths such as hope, persistence, and leadership not only has the potential to improve general self-efficacy but may also translate into higher levels of academic self-efficacy, which is crucial for students’ academic performance and success.

One of the main strengths of this study lies in its comprehensive approach to the relationships between character strengths and general and academic self-efficacy. Including mediation analysis, it allows us to identify the crucial role that general self-efficacy plays in the relationship between character strengths and academic self-efficacy, providing a more nuanced perspective on the underlying mechanisms in these processes. Among the theoretical implications, researchers highlight a notable connection to the findings of the engine model of positive schooling (Harzer et al., 2021), which views character strengths as inputs that influence educational outcomes mediated by psychological processes such as self-efficacy. Additionally, studies have found that academic self-efficacy mediates alongside character strengths and academic achievements (Weber and Harzer, 2022). One example of these outcomes could be academic performance, although the current study did not measure this aspect.

Another theoretical implication is the relevance of the Social Cognitive Career Theory in explaining the fundamental role of self-efficacy as a precursor to outcome expectations in achieving personal goals within academic and professional contexts. This model integrates personal and contextual variables constantly interacting with the behavioral self-system. Therefore, the cognitive variables in the model do not operate in a vacuum; they relate to personality and the real-world environment, which are considered contextual variables. These factors act as moderators in the relationship between goals and career planning. In this sense, character strengths are considered personal variables that could influence the core of the model, which is a relevant aspect that deserves greater attention in future studies, as the scientific literature still needs to be explored, (Lent and Brown, 2013; Wendling and Sagas, 2020).

Another strength is that it constitutes a study outside the Western-, Educated-, Industrialized-, Rich-, and Democratic- (WEIRD) cultural context, serving as evidence of the application of positive psychology while considering other cultural and linguistic contexts (Duan et al., 2022). Therefore, the principal value of this study lies in delving into the relationship of these variables in a large sample of university students in Venezuela, a Latin American country. Despite its strengths, this study presents some limitations that should be considered. First, the correlational nature of the research prevents establishing causal relationships between the studied variables. Although significant associations were identified, it cannot be definitively concluded that character strengths cause an increase in self-efficacy. Additionally, the sample consisted exclusively of Venezuelan university students, which may limit the generalizability of the findings to other populations and cultural contexts. Another limitation is self-report measures, which may be subject to social desirability biases or self-reporting errors.

The findings of this study have significant implications for the educational field. They underscore the relevance of character strengths, such as hope, persistence, and leadership, in promoting academic self-efficacy. This suggests that educational interventions aimed at strengthening these characteristics could effectively enhance students’ confidence and academic performance. Furthermore, the mediating role of general self-efficacy implies that strategies to enhance general perceptions of self-efficacy could also positively impact academic self-efficacy. This is particularly relevant for support and counseling programs in the university setting, which could use orientation programs, mentoring, and psychological counseling to help students develop a positive perception of their abilities in general, thereby positively impacting their academic performance.

The findings also imply that curricula and teaching methods should be designed to impart knowledge and strengthen the mentioned characteristics. For example, tasks and projects can be structured in a way that requires perseverance, fosters hope for future success, and develops leadership skills, thus preparing students for both academic and professional challenges. Alongside the above, implementing continuous assessments and personalized feedback could focus on academic performance and developing these character strengths. By providing feedback that reinforces behaviors associated with hope, persistence, and leadership, educators can help students recognize and cultivate these strengths. These implications suggest that a more comprehensive and holistic approach to developing students’ character can impact their academic confidence and success in higher education.

Future research could address some of these limitations by using longitudinal or experimental designs that examine causality in the relationships between character strengths and self-efficacy. Additionally, it would be valuable to replicate this study in different cultural contexts and with more diverse samples to assess the generality of the findings. Because in this study data collection was done through self-reports, a strategy that, although useful to capture the subjective perception of the participants, may limit the objectivity of the results due to self-report bias or social desirability, corresponding to a limitation in this research. Therefore, future research could consider peer evaluations and behavioral observations to obtain a more comprehensive view of the variables under study. Another limitation lies in the use of non-probabilistic sampling, which affects the generalization of the results to other samples and other cultural and academic contexts. Future research could also explore specific interventions designed to strengthen the characteristics identified as most influential on self-efficacy and evaluate their effectiveness in improving academic performance and overall student well-being. Finally, it would be interesting to investigate how other variables, such as social support or well-being, interact with character strengths and self-efficacy in the educational context.

## 5 Conclusion

This study demonstrates that character strengths such as hope, persistence, and leadership are significant predictors of general and academic self-efficacy in this sample of university students in Venezuela. General self-efficacy partially mediates the relationship between these strengths and academic self-efficacy, suggesting that fostering these qualities can enhance the university student’s experience. These findings underscore the importance of integrating educational programs that strengthen these characteristics to optimize agency and academic success in the university context.

## Data Availability

The raw data supporting the conclusions of this article will be made available by the authors, without undue reservation.
